# Structural Features of the Temporomandibular Joint Evaluated by MRI and Their Association with Oral Function and Craniofacial Morphology in Female Patients with Malocclusion: A Cross-Sectional Study

**DOI:** 10.3390/jcm14144921

**Published:** 2025-07-11

**Authors:** Mari Kaneda, Yudai Shimpo, Kana Yoshida, Rintaro Kubo, Fumitaka Kobayashi, Akira Mishima, Chinami Igarashi, Hiroshi Tomonari

**Affiliations:** 1 Department of Orthodontics, Tsurumi University School of Dental Medicine, Yokohama 230-8501, Japan; kaneda-m@tsurumi-u.ac.jp (M.K.); yoshidakana0x0@gmail.com (K.Y.); rin.k.regulus@gmail.com (R.K.); fkobayashi.tsu@gmail.com (F.K.); tomonari-h@tsurumi-u.ac.jp (H.T.); 2 Department of Diagnostic Imaging, Tsurumi University Dental Hospital, Yokohama 230-8501, Japan; 3 Department of Oral and Maxillofacial Radiology and Diagnosis, Tsurumi University School of Dental Medicine, Yokohama 230-8501, Japan; igarashi-c@tsurumi-u.ac.jp

**Keywords:** temporomandibular joint disorders, magnetic resonance imaging, DC/TMDs, female patients, occlusal function, craniofacial morphology, malocclusion, joint displacement, osteoarthritis

## Abstract

**Background/Objectives**: Temporomandibular disorders (TMDs) are a group of musculoskeletal and neuromuscular conditions involving the temporomandibular joint (TMJ), masticatory muscles, and related anatomical structures. Although magnetic resonance imaging (MRI) is considered a noninvasive and highly informative imaging modality for assessing TMJ soft tissues, few studies have examined how TMJ structural features observed on MRI findings relate to oral function and craniofacial morphology in female patients with malocclusion. To investigate the associations among TMJ structural features, oral function, and craniofacial morphology in female patients with malocclusion, using MRI findings interpreted in conjunction with a preliminary assessment based on selected components of the DC/TMDs Axis I protocol. **Methods**: A total of 120 female patients (mean age: 27.3 ± 10.9 years) underwent clinical examination based on DC/TMDs Axis I and MRI-based structural characterization of the TMJ. Based on the structural features identified by MRI, patients were classified into four groups for comparison: osteoarthritis (OA), bilateral disk displacement (BDD), unilateral disk displacement (UDD), and a group with Osseous Change/Disk Displacement negative (OC/DD (−)). Occlusal contact area, occlusal force, masticatory efficiency, tongue pressure, and lip pressure were measured. Lateral cephalometric analysis assessed skeletal and dental patterns. **Results**: OA group exhibited significantly reduced occlusal contact area (*p* < 0.0083, η^2^ = 0.12) and occlusal force (*p* < 0.0083, η^2^ = 0.14) compared to the OC/DD (−) group. Cephalometric analysis revealed that both OA and BDD groups had significantly larger ANB angles (OA: 5.7°, BDD: 5.2°, OC/DD (−): 3.7°; *p* < 0.0083, η^2^ = 0.21) and FMA angles (OA: 32.4°, BDD: 31.8°, OC/DD (−): 29.0°; *p* < 0.0083, η^2^ = 0.17) compared to the OC/DD (−) group. No significant differences were observed in masticatory efficiency, tongue pressure, or lip pressure. **Conclusions**: TMJ structural abnormalities detected via MRI, especially osteoarthritis, are associated with diminished oral function and skeletal Class II and high-angle features in female patients with malocclusion. Although orthodontic treatment is not intended to manage TMDs, MRI-based structural characterization—when clinically appropriate—may aid in treatment planning by identifying underlying joint conditions.

## 1. Introduction

Temporomandibular disorders (TMDs) are a group of musculoskeletal and neuromuscular conditions involving the temporomandibular joint (TMJ), masticatory muscles, and related anatomical structures. These conditions present with a range of clinical symptoms, including joint pain, joint sounds (e.g., clicking or crepitus), muscle tenderness, and restricted mandibular movement, which may significantly impair oral function and quality of life [1,2]. TMDs are widely recognized as multifactorial disorders involving structural, functional, psychological, hormonal, and behavioral components [3,4,5,6].

Epidemiological studies and meta-analyses report that TMDs affect approximately 10–15% of the general population, with a markedly higher prevalence in females and young adults [7]. Several biological and behavioral factors—including hormonal differences, psychological stress, and parafunctional behaviors—have been suggested as contributors to these demographic disparities [8]. For this reason, female participants are frequently prioritized in clinical studies of TMDs.

With respect to morphological characteristics, some observational studies have suggested a potential association between TMDs and specific craniofacial or occlusal features, such as skeletal Class II and III patterns, deep bite, and open bite [9,10,11,12,13,14]. However, recent systematic reviews have criticized earlier research for methodological limitations, including selection bias and insufficient control groups, and have emphasized that the causal relationship between malocclusion and TMDs remains inconclusive [15,16].

It is clinically intuitive that orofacial pain and joint dysfunction may contribute to reduced occlusal force and masticatory performance. Empirical studies have attempted to quantify these impairments using objective functional metrics, and their findings suggest that pain-related inhibition and altered muscle coordination may underlie decreased oral function in individuals with TMD [17,18,19]. However, due to heterogeneity in methodology and inconsistencies in outcome definitions across studies, these results should be interpreted with caution.

To account for the multifactorial nature of temporomandibular disorders (TMDs), the Diagnostic Criteria for Temporomandibular Disorders (DC/TMDs) was developed as a standardized system for both clinical and research purposes [20,21]. This framework consists of two complementary axes: Axis I focuses on physical diagnoses such as disk displacement, myofascial pain, and osteoarthritis, while Axis II assesses psychosocial factors, including pain-related disability and psychological distress. Despite its widespread adoption, the DC/TMDs protocol relies primarily on patient interviews and clinical examinations, which may have limitations in detecting specific structural conditions of the TMJ, especially in the absence of overt symptoms [22]. In this context, magnetic resonance imaging (MRI) may serve as a supplemental modality to characterize the structural features of the TMJ in research settings. However, it is not our intention to redefine asymptomatic individuals as patients based solely on imaging findings. Instead, we emphasize that MRI-based structural assessments should be interpreted independently of Axis I diagnoses and should not be equated with diagnostic categorization. Importantly, although the present study focuses on structural evaluation, recognition of Axis II factors remains essential in the comprehensive assessment of TMDs.

As a complementary tool, magnetic resonance imaging (MRI) is widely regarded as a noninvasive and informative modality for assessing both the soft and hard tissues of the TMJ, particularly the position and morphology of the articular disk, as well as joint effusion and osseous changes such as bone marrow edema and condylar deformation [23,24,25,26]. MRI-based three-dimensional assessment can provide detailed visualizations of TMJ structures, potentially enhancing our understanding of joint morphology and its variation among individuals with skeletal malocclusion. However, the presence of such findings does not necessarily indicate a pathological condition or a causal link to TMD symptoms, as similar structural variations have been observed in asymptomatic individuals [27,28]. Therefore, MRI-observed features should be interpreted cautiously and in conjunction with clinical findings, particularly to avoid misclassification or overtreatment.

Despite advances in diagnostic imaging, few studies have explored how structural features of the temporomandibular joint (TMJ) observed via MRI—when interpreted alongside a preliminary assessment based on selected components of the DC/TMD Axis I protocol—relate to craniofacial morphology in patients with malocclusion [27,28]. Moreover, to our knowledge, no previous study has simultaneously evaluated both craniofacial morphology and oral function in this population within a standardized clinical and imaging-based framework. Given the higher prevalence of TMDs among females [7], and in order to reduce sample heterogeneity, the present study focuses exclusively on female patients. This design choice was intended to improve internal validity by examining the potential associations between TMJ structural variation and the functional or morphological characteristics within the subgroup most commonly affected by TMDs. However, we fully recognize the importance of including male patients in future studies to assess sex-related differences in TMJ structure and function.

Therefore, the purpose of this cross-sectional study is to investigate the relationships among MRI-based structural features of the temporomandibular joint (TMJ), oral function, and craniofacial morphology in female patients with malocclusion. Structural conditions of the TMJ were assessed using magnetic resonance imaging (MRI), with imaging findings interpreted in parallel with a preliminary clinical screening based on selected components of the DC/TMDs Axis I protocol. This study specifically aims to compare oral function and craniofacial morphology across TMJ condition groups classified by DC/TMDs and supported by MRI, and to explore the potential associations between morphological and functional variables. By integrating MRI-based structural characterization, objective evaluations of oral function, and cephalometric analysis, we sought to obtain a comprehensive understanding of the structural and functional features that may accompany TMJ variation in this population. We hypothesized that patients with MRI-detected TMJ structural alterations would exhibit diminished oral function and distinct craniofacial morphology compared to those without such findings.

## 2. Materials and Methods

### 2.1. Subjects, Eligibility Criteria, and Group Setting

**Figure 1:**
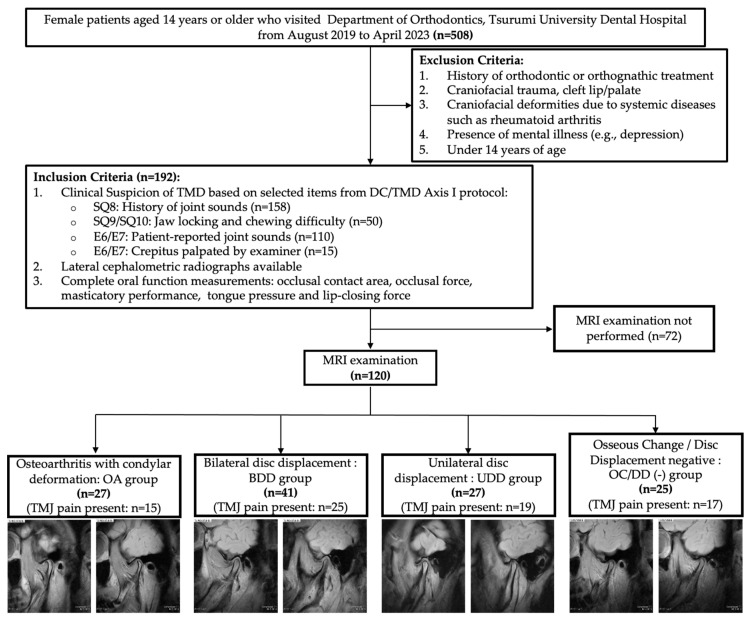
Flowchart illustrating subject recruitment and representative MRI images depicting typical structural features for each group.

This observational study was conducted in accordance with the STROBE (Strengthening the Reporting of Observational Studies in Epidemiology) guidelines [29]. Among 508 female patients aged 14 years or older who visited the Department of Orthodontics, Tsurumi University Dental Hospital, from August 2019 to April 2023, 120 female patients (mean age: 27.3 ± 10.9 years) who met the inclusion criteria and agreed to undergo MRI were included. The patient selection flow is shown in Figure 1.

Inclusion Criteria:

(1) Clinical suspicion of TMD based on selected items from the English version of the DC/TMDs Axis I protocol [20]. Specifically, patients were considered “suspected TMD” cases if they responded affirmatively to one or more of the following items:
  – SQ8: History of temporomandibular joint (TMJ) sounds (e.g., clicking).
  – SQ9 and/or SQ10: Episodes of jaw locking or difficulty in chewing.
  – E6 and/or E7: Patient-reported TMJ sounds and/or crepitus palpated by the examiner during mandibular movement.

This limited clinical screening was used as a preliminary assessment to identify candidates for MRI referral.

(2) Availability of lateral cephalometric radiographs.
(3) Complete oral function measurements: occlusal contact area, occlusal force, masticatory performance, tongue pressure, lip-closing force.

Exclusion Criteria:

(1) History of orthodontic or orthognathic treatment.
(2) Craniofacial trauma, cleft lip/palate.
(3) Craniofacial deformities due to systemic diseases such as rheumatoid arthritis.
(4) Presence of mental illness (e.g., depression).
(5) Age under 14 years.

Group classification (based on MRI findings):

OA group: Osteoarthritis with condylar deformation group.
BDD group: Bilateral disk displacement group.
UDD group: Unilateral disk displacement group.
OC/DD (−) group: Osseous Change/Disc Displacement negative group.

### 2.2. MRI Examination and Diagnosis

MRI scans were obtained as a supplemental imaging method, used when clinically indicated, and to confirm the diagnosis obtained with the DC/TMD, using a 0.4T open MRI system (APERTO Inspire, Fujifilm Medical, Tokyo, Japan) with a 10.0 cm TMJ surface coil. The MRI protocol for the temporomandibular joint included the following four sequences: (1) sagittal view at the intercuspal position (T2-star) with scanning parameters of repetition time (TR) = 400 ms, echo time (TE) = 14 ms, flip angle = 45°, bandwidth = 8.0 kHz, matrix size = 256 × 192, field of view (FOV) = 120 mm, slice thickness = 4 mm, number of slices = 9 (per side) and scan duration = 5 m 9 s (per side); (2) coronal view at the intercuspal position (T2-star) with TR = 400 ms, TE = 14 ms, flip angle = 45, bandwidth = 10.0 kHz, matrix size = 256 × 192, field of view (FOV) = 120 mm, slice thickness = 4 mm, number of slices = 9 (per side) and scan duration = 5 m 23 s (left and right, respectively); (3) sagittal T2 image with TR = 4400 ms, TE = 100 ms, flip angle = 90°, bandwidth = 12.5 kHz, matrix size = 192 × 192, FOV = 120 mm, slice thickness = 5 mm, number of slices = 6 (per side) and scan duration = 3 m 36 s (per side); and (4) sagittal view at the open-mouth position (T1) with TR = 1100 ms, TE = 30 ms, flip angle = 90°, bandwidth = 36.5 kHz, matrix size = 256 × 192, FOV = 120 mm, slice thickness = 5 mm, number of slices = 12 (in total) and scan duration = 3 m 33 s. All sequences were obtained with a field of view (FOV) of 120 mm, which ensured complete coverage of the temporomandibular joint region bilaterally. During MRI acquisition, patients were positioned in the supine position. For the closed-mouth position, patients were instructed to maintain their natural occlusion. For the open-mouth position, a bite block was used to support the mandible and maintain a stable open position during scanning.

These images were acquired by a certified radiologic technologist at the Department of Diagnostic Imaging (chief technologist: A.M), Tsurumi University Dental Hospital, and were interpreted to characterize disk position and condylar shape by a board-certified oral radiologist at the Department of Oral and Maxillofacial Radiology and Diagnosis (chief professor: C.I), Tsurumi University School of Dental Medicine, in accordance with established previously described MRI-based structural criteria [30,31,32,33,34]. Although the MRI examinations in this study were performed using a 0.4T open MRI system, this protocol has been used in previous clinical TMJ studies conducted at our institution and has shown acceptable diagnostic reliability for identifying disk displacement and osseous changes in the TMJ [34].

### 2.3. Evaluation of Oral Function

Evaluation methods of oral function have been described elsewhere [35]. Five parameters were assessed: occlusal contact area, maximum occlusal force, masticatory performance, tongue pressure, and lip pressure. Each was measured three times by trained examiners, and the average was used for analysis.

#### 2.3.1. Occlusal Contact Area and Occlusal Force

Occlusal contact area and occlusal force were assessed using Dental Prescale II (GC Corporation, Tokyo, Japan) [36]. Subjects practiced biting on a training film inserted intraorally for 3 s with their molars. Subsequently, the actual measurement was conducted using the Prescale film. A specialized scanner was used to analyze the film and calculate the contact area and maximum occlusal force.

#### 2.3.2. Masticatory Performance

Masticatory efficiency was assessed using color-changeable chewing gum (Chew Check Gum, Oral Care Inc., Tokyo, Japan) [37]. After rinsing for 15 s, subjects chewed the gum for 60 cycles and then flattened it into a circular shape. The color change of the gum was used as an indicator of masticatory efficiency and measured using a colorimeter (CR-20, Konica Minolta Inc., Tokyo, Japan). Measurements were performed three times each in the order of free chewing, right-side chewing, and left-side chewing, with 3 min rest periods between each chewing trial. The mean value of free chewing was used for analysis.

#### 2.3.3. Tongue Pressure

Tongue pressure was evaluated using the JMS tongue-pressure measuring device (JMS Co. Ltd., Hiroshima, Japan) [38]. Subjects were instructed to press their tongue firmly against the palate for 7 s with the probe placed on the tongue. After one practice attempt, three measurements were taken, and the average was recorded as tongue pressure.

#### 2.3.4. Lip Pressure

Lip pressure was measured using the Ripple-Kun device (Shofu Inc., Tokyo, Japan) [39]. Subjects were instructed to insert the button into the oral vestibule and close their lips. The button was then pulled horizontally, and the force at which it exited the oral vestibule was measured. Following one practice trial, three measurements were taken, and the average was calculated as lip pressure.

#### 2.3.5. Reliability

Calibration was performed before use, and tests were conducted by trained examiners. Intra-class correlation coefficients (ICC) were 0.73 for masticatory performance, 0.95 for occlusal force, 0.86 for contact area, 0.83 for lip pressure, and 0.96 for tongue pressure [35].

### 2.4. Evaluation of Craniofacial Morphology

**Figure 2:**
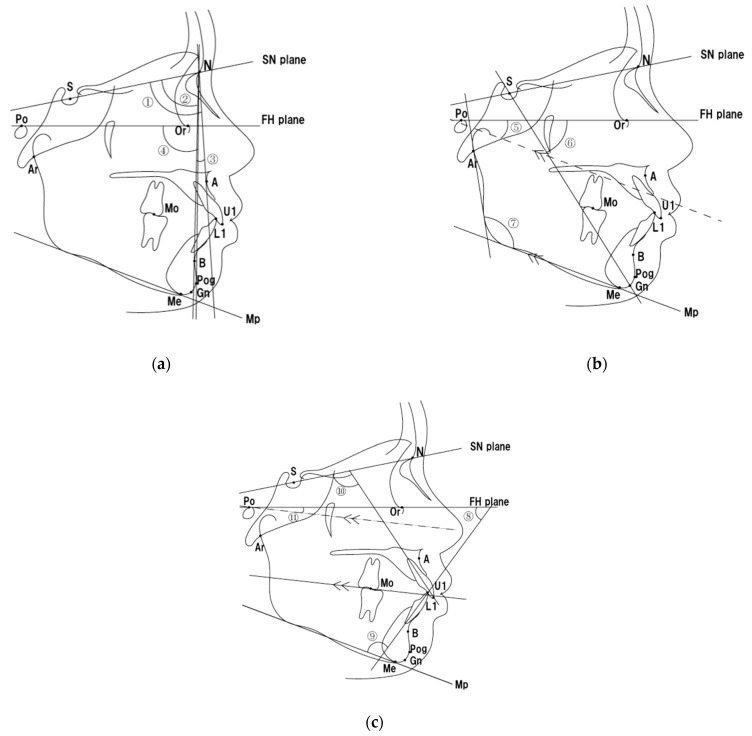
Anatomical landmarks, points, and angles: (**a**) antero-posterior skeletal pattern; (**b**) vertical skeletal pattern; (**c**) dental pattern.

Lateral cephalograms were taken with ear rods using a CX-150ST 8000C device (ASAHIROENTGEN IND. Co., Ltd., Kyoto, Japan). Settings: 150 kV; 250 mA; 0.32 s; FFD: 1650 mm. Tracings were digitized and analyzed using WinCeph ver.11.0 (Rise Co., Ltd., Miyagi, Japan). The anatomical landmarks, points, and angles used as endpoints in this study are shown in Table 1 and Figure 2. Linear measurements of overjet (OJ) and overbite (OB) were performed using dental models and calipers. The following 13 items are measured:

Antero-posterior skeletal patterns: ① SNA, ② SNB, ③ ANB, ④ Facial angle.

Vertical skeletal patterns: ⑤ FMA, ⑥ Y-axis, ⑦ Gonial angle.

Dental pattern: ⑧ FMIA, ⑨ IMPA, ⑩ U1 to SN, ⑪ Occlusal plane to FH, ⑫ Overjet, ⑬ Overbite.

All measurements were performed by a single orthodontist at Department of Orthodontics, Tsurumi University School of Dental Medicine (M.K). Intra-class correlation coefficients (ICCs) were confirmed by tracing data from 10 subjects twice with a 2-week interval, and ICC > 0.95 was confirmed. Measurement methods and reliability assessment followed the method of Xiong et al. (2020) [40].

### 2.5. Sample Size Calculation

Sample size estimation was performed using G*Power (version 3.1.9.7) for one-way ANOVA with four independent groups. Based on effect size benchmarks specific to temporomandibular joint (TMJ) research, as proposed by Zieliński and Gawda (2025) [41], a large effect size was anticipated. According to their study, comparisons involving joint structural differences and masticatory function typically yield large effect sizes (Hedges’ g ≈ 0.70), which corresponds to Cohen’s f ≈ 0.40. Assuming an alpha level of 0.05, power of 0.90, and effect size f = 0.40, the required sample size was calculated to be 96. To account for potential data exclusions, a total of 120 participants were enrolled. This sample size is considered sufficient to detect clinically meaningful differences among the groups.

### 2.6. Statistical Analysis

Normality was confirmed using the Kolmogorov–Smirnov test, Shapiro–Wilk test, and histogram inspection (Table S1 and Figure S1). As the data were assumed to be normally distributed, one-way ANOVA was used to compare variables among the four groups. To control for type I error due to multiple comparisons, Bonferroni correction was applied in the post hoc analysis [42]. Accordingly, the threshold for statistical significance was adjusted to *p* < 0.0083 (0.05/6), based on six pairwise group comparisons (OA vs. BDD, OA vs. UDD, OA vs. OC/DD (−), BDD vs. UDD, BDD vs. OC/DD (−), UDD vs. OC/DD (−)). For each ANOVA, effect sizes were calculated using Eta squared (η^2^). Effect sizes were interpreted as follows: η^2^ = 0.01 (small), η^2^ = 0.06 (medium), and η^2^ = 0.14 (large). While some comparisons reached statistical significance, those with small effect sizes (η^2^ < 0.06) were not considered clinically meaningful in accordance with established interpretation criteria [43]. All analysis were performed using SPSS Statistics ver27.0 (IBM, Tokyo, Japan).

## 3. Results

### 3.1. Demographic and Clinical Characteristics

Figure 1 shows the number of subjects in each group: 27 subjects in the OA group, 41 subjects in the BDD group, 27 subjects in the UDD group, and 25 subjects in the OC/DD (−) group, along with representative MRI images illustrating the structural characteristics typically observed in each group. No significant differences in age were found among the four groups (Table 2).

### 3.2. TMJ Pain Prevalence

Table 2 shows the prevalence of TMJ pain in each group. No significant differences were observed in the prevalence of pain among the four groups (*p* > 0.05). Notably, a high proportion of TMJ pain was also reported in the OC/DD (−) group (68%), suggesting that TMJ pain may not always be associated with identifiable structural characteristics in MRI.

### 3.3. Comparison of Oral Function

Table 3 shows the results of intergroup comparisons of oral function. Occlusal contact area was significantly lower in OA (10.36 ± 4.39 mm^2^) compared to OC/DD (−) (18.63 ± 10.6 mm^2^) group (*p* < 0.0083). Additionally, maximum occlusal force was significantly lower in the OA (429.1 ± 151.77 N) compared to the OC/DD (−) (708.52 ± 368.55 N) group (*p* < 0.0083). No significant differences were observed among groups for masticatory performance, tongue pressure, or lip pressure.

### 3.4. Comparison of Craniofacial Morphology

#### 3.4.1. Antero-Posterior and Vertical Skeletal Patterns

Table 4 shows the results of cephalometric analysis across four groups. The OA and BDD groups had significantly larger ANB angles (OA: 4.43 ± 2.85°, BDD: 3.27 ± 3.73°, OC/DD (−): 0.31 ± 2.83°) and significantly smaller SNB angles (OA: 76.73 ± 4.7°, BDD: 77.41 ± 5.05°, OC/DD (−): 81.67 ± 3.93°), suggesting a skeletal Class II tendency (*p* < 0.0083). FMA angles were significantly larger in the OA and BDD groups (OA: 34.81 ± 7.44°, BDD: 32.79 ± 8.0°, OC/DD (−): 25.71 ± 7.31°), suggesting a high-angle tendency (*p* < 0.0083). Other parameters, such as Facial angle and Y-axis, also tended to indicate a skeletal Class II tendency in the OA group.

#### 3.4.2. Comparison of Dental Morphology

As shown in Table 4, the OA group showed significantly smaller U1 to SN angles compared to the OC/DD (−) group (OA: 104.56 ± 6.39°, OC/DD (−): 113.06 ± 9.25°) and significantly larger Occlusal plane to FH angles compared to the OC/DD (−) group (OA: 20.06 ± 4.91°, OC/DD (−): 14.78 ± 4.61°) (*p* < 0.0083). The BDD groups showed significantly larger Overjet compared to the OC/DD (−) group (BDD: 5.22 ± 4.15 mm, OC/DD (−): 1.88 ± 3.52 mm) (*p* < 0.0083).

## 4. Discussion

This cross-sectional study examines the relationship between temporomandibular joint (TMJ) structural features observed via MRI and oral function and craniofacial morphology in female patients with malocclusion. Patients were categorized into four groups based on MRI-identified structural conditions, interpreted in conjunction with DC/TMD-based clinical screening. Our findings indicate that patients with osteoarthritis (OA)-like structural changes exhibit significantly reduced occlusal contact area and occlusal force compared to those without observable structural abnormalities (OC/DD (−) group). These results suggest that TMJ structural abnormalities, particularly condylar deformities, may be associated with diminished occlusal support function.

This observation is in line with prior studies. For example, Bavia et al. reported associations between facial morphology and occlusal force in patients with TMD [44]. The current findings extend this knowledge by linking MRI-based joint features to functional and morphological outcomes. Interestingly, patients with unilateral disk displacement (UDD group) did not show significant reductions in oral function, aligning with reports by Sato et al., who found no marked decrease in occlusal force or masticatory performance in cases of unilateral joint abnormalities [45]. This emphasizes the potential functional impact of bilateral TMJ involvement. In our study, MRI served as a supplementary imaging modality and was not employed as a standalone diagnostic tool. Interpretation of the findings was guided by previously validated imaging protocols [23,24,25,26,27,28]. While TMJ pain was assessed, we did not evaluate pain intensity or disability using validated Axis II instruments. Furthermore, variables such as masseter muscle thickness, which may influence occlusal function, were not assessed. These limitations should be addressed in future investigations.

Notably, no significant differences were found among the groups in terms of masticatory efficiency, tongue pressure, or lip pressure. The chewing efficiency test used in this study, which relies on the color change in chewing gum, mainly evaluates mixing ability rather than crushing performance. Previous research by Sasaki et al., Lepley et al., and Mizokami et al. suggested that mixing ability and crushing ability represent distinct muscle activities [35,46,47]. Tomonari et al. also reported that muscle activity varies according to the hardness of the test food [48]. These insights imply that the soft chewing gum used in this study may not detect subtle functional differences. Furthermore, Schimmel et al. found no clear correlation between occlusal force and masticatory efficiency [49], supporting our results. Moreover, Sasaki et al. reported significant correlations between masticatory performance and both tongue and lip pressure [35], indicating that soft tissue functions significantly influence mixing ability. While the color-changeable chewing gum test provides an accessible method for assessing mixing ability, its correlation with occlusal force is limited. Solid food-based tests (e.g., carrots or almonds) may provide more accurate evaluations of masticatory efficiency, particularly with respect to crushing ability.

Tongue and lip pressure are produced by the tongue and orbicularis muscle, respectively, and differ from occlusal support muscle activity. Additionally, these tests measure static maximal strength, not dynamic aspects such as endurance or coordination during mastication and swallowing. Hence, further studies using electromyography (EMG) and foods with different textures may provide more comprehensive assessments of specific oral motor functions relevant to mastication.

Cephalometric analysis revealed that the OA and BDD groups exhibited features suggestive of skeletal Class II and high-angle morphology, including significantly larger ANB and FMA angles and smaller SNB angles, compared to the OC/DD (−) group. The OA group also showed dental compensations, such as lingual inclination of the maxillary incisors, as indicated by a significantly smaller U1 to SN angle than both the OC/DD (−) group and previously reported Japanese female norms (approximately 107.8°) [50]. While these findings suggest structural variation in mandibular position and vertical dimension, the cross-sectional design limits causal inferences. Although no control group without malocclusion was included, reference values from Japanese females with Class I malocclusion (e.g., ANB ~ 3.3°) indicate that the mean ANB value in the OA group (4.43°) reflects a Class II tendency beyond typical malocclusion norms [50]. Previous observational studies have reported similar associations between TMJ symptoms and craniofacial morphology [19,51,52,53,54], although the directionality and clinical implications remain uncertain. These findings may support the hypothesis that underlying craniofacial patterns and TMJ structural findings often coexist in malocclusion cases, rather than indicating a direct causal relationship.

In terms of joint pain, there were no significant differences in prevalence among the four groups. This may reflect limitations in the DC/TMD Axis I assessment of pain, which focuses on pain history within the past 30 days and provoked responses [20], without capturing the broader psychosocial context of pain. In contrast, validated pain intensity and disability instruments, such as the Visual Analog Scale (VAS), Numerical Rating Scale (NRS), Jaw Functional Limitation Scale (JFLS), and Quantitative Sensory Testing (QST), offer more nuanced assessments of pain experience and functional impact [55,56,57,58]. The absence of such Axis II-oriented measures in this study limits our interpretation of the pain-related findings. Notably, the relatively high prevalence of TMJ pain in the OC/DD (−) group underscores that joint pain may occur in the absence of structural abnormalities, and could reflect psychosocial or neuromuscular contributors. Future studies should incorporate comprehensive Axis II assessments to clarify the multifactorial nature of TMJ pain and its interaction with structural and functional parameters. Furthermore, experimental studies have shown that induced orofacial pain does not always reduce occlusal force or masticatory efficiency, supporting the need for a multidimensional framework in interpreting these relationships [59,60,61].

While our findings suggest that certain TMJ structural features, particularly osteoarthritic changes and bilateral disk displacement, may be associated with reduced occlusal function and high-angle skeletal morphology in female patients with malocclusion, these observations should not be interpreted as evidence of causality or diagnostic necessity. MRI can serve as a supplemental tool in complex cases, but its role in routine orthodontic treatment planning remains limited and must be considered alongside clinical symptoms and psychosocial factors.

However, some limitations should be noted. As a cross-sectional study, causal relationships cannot be inferred. The sample consisted only of females and included only patients suspected of TMD, which may introduce selection bias. Furthermore, we did not include a control group of individuals without malocclusion, which limits our ability to determine whether the observed TMJ findings are exclusive to malocclusion. Importantly, this study focuses only on MRI-detectable TMJ structural features and does not incorporate assessments of myofascial or psychosocial contributors to TMD. Interestingly, a high pain prevalence was observed even in the OC/DD (−) group (68%), which lacked apparent structural abnormalities. This underscores that TMJ pain can arise in the absence of structural changes, likely due to multifactorial influences including psychosocial stress, central sensitization, or muscle-related dysfunction. As this study did not assess Axis II (psychosocial) components, potential confounding factors could not be evaluated. Future studies should incorporate DC/TMDs Axis II assessments, longitudinal designs, and CBCT-based three-dimensional evaluations to clarify the contribution of psychosocial and non-structural factors to the multifactorial nature of TMDs.

A key limitation of this study is the use of a 0.4T open MRI system, which may have lower sensitivity compared to higher-field systems (e.g., 1.5T or 3T) for detecting subtle structural changes, particularly in early-stage TMDs. Although the imaging protocol was based on prior research conducted at our institution, caution is warranted in interpreting findings where fine detail may be diagnostically significant. Additionally, the slice thickness used in this study (4–5 mm) may have limited spatial resolution, potentially leading to the underdetection of small lesions or subtle TMJ abnormalities. This parameter was determined based on the technical capabilities of the 0.4T MRI system used, but we acknowledge that thinner slices would provide better anatomical detail. Moreover, 3D sequences were not employed, which may have limited diagnostic precision in detecting complex disk displacements. These imaging limitations may partially explain the challenges of detecting early-stage structural changes in Axis I diagnoses and underscore the need for multimodal assessment strategies.

Although Bonferroni correction was applied to control for multiple-group comparisons per variable, no global adjustment was conducted across the 13 cephalometric variables. As such, the possibility of inflated Type I error due to multiple-endpoint testing cannot be entirely excluded. Additionally, cephalometric parameters such as ANB showed statistically significant differences across groups, but the clinical significance of relatively small angular differences (e.g., 2–3°) should be interpreted with caution. These findings, while statistically significant, should not be taken as evidence for diagnostic categorization or treatment indication without further clinical validation. Future studies comparing against normative clinical thresholds may help clarify their relevance for treatment planning.

The method used for assessing masticatory efficiency, based on color-changeable chewing gum, has not been validated against gold-standard techniques such as sieving or spectrophotometry. Furthermore, the ICC value of 0.73 for this test indicates moderate reliability, which may have introduced variability in the measurements. Masticatory performance was evaluated using a color-changeable chewing gum, which may not reflect crushing ability or masticatory performance for solid foods. Tongue-pressure and lip-closing force measurements in this study focused on static muscle strength and did not reflect dynamic functional tasks such as swallowing, speech, or continuous oral coordination. This may limit the interpretation of oral function in real-life conditions. The lack of differences in tongue and lip pressure among groups may be due to methodological constraints. Static measurements may not capture dynamic function, such as atypical swallowing. Sasaki et al. (2023) reported correlations between tongue pressure and malocclusion [35], suggesting that further studies using dynamic tests or EMG are warranted. These functional assessments should be viewed as exploratory tools to characterize physiological trends, rather than as definitive clinical indicators.

## 5. Conclusions

The MRI-observed structural features of the temporomandibular joint (TMJ)—particularly condylar deformities and bilateral disk displacement—were associated with reduced occlusal function and a skeletal Class II high-angle pattern in female patients with malocclusion. These findings suggest that TMJ morphology may contribute to variations in oral function and craniofacial form. However, due to the multifactorial etiology of temporomandibular disorders, structural changes should be interpreted in conjunction with clinical findings and psychosocial assessments. MRI may serve as a supplemental tool in complex cases, but its routine use in orthodontic diagnostics warrants careful consideration. Further longitudinal multi-dimensional studies incorporating the DC/TMDs Axis II and 3D imaging are essential to elucidate the clinical significance of these structural findings and their implications for orthodontic treatment planning.

## Figures and Tables

**Table 1:** Anatomical landmarks, points, and angles.

Landmarks and Points	
S (sella)	Center of sella turcica
N (nasion)	The most anterior point of the frontonasal suture
Or (orbitale)	The lowest point on the average left and right inferior borders of the bony orbit
Po (porion)	The highest point on the superior surface of the soft tissue of the external auditory meatus
A-Point	The most posterior point on the anterior contour of the upper alveolar process
B-Point	The most posterior point on the anterior contour of the lower alveolar process
Me (menton)	The lowest point on the mandibular symphysis
Pog (Pogonion)	The most anterior point of the midline cross-sectional image of the mandibular miter
Gn (gnathion)	The midpoint of the nasolabial groove
Ar (articulare)	The intersection of the posterior margin of the mandibular branch and subnasal margin of the occipital bone
U1	The tip of the maxillary central incisor
L1	The tip of the mandibular central incisor
Mo	The central point of the cuspid fit of the upper and lower first molars
Angles	
① SNA (°)	The angle between the SN plane and NA line
② SNB (°)	The angle between the SN plane and NB line
③ ANB (°)	The angle formed by point A, nasion, and point B
④ Facial angle (°)	The angle between the Frankfurt (FH; Or-Po) plane and N-Pog plane
⑤ FMA (°)	The angle the between Mandibular inferior margin plane (Mp) and the FH plane
⑥ Y-axis (°)	The angle between the Frankfurt (FH; Or-Po) plane and S-Gn plane
⑦ Gonial angle (°)	The angle the between Mandibular inferior margin plane (Mp) and the ramus plane
⑧ FMIA (°)	The angle between the Frankfurt (FH; Or-Po) plane and the long axis of the L1
⑨ IMPA (°)	The angle between the long axis of the L1 and the Mandibular inferior margin plane (Mp)
⑩ U1 to SN (°)	The angle between the long axis of the U1 and the SN plane
⑪ Occlusal plane to FH (°)	The angle between the line connecting Mo and midpoints of U1 and L1 and the FH plane

**Table 2:** Median age and prevalence of TMJ pain across groups classified by MRI-observed structural features.

Group	Age	TMJ Pain Present (n)	TMJ Pain Absent (n)	Total (n)	Pain Prevalence (%)
OA	24 (17.5–30.5)	15	12	27	55.6
BDD	25 (18–32)	25	16	41	61
UDD	22 (14.5–29.5)	19	8	27	70.4
OC/DD (−)	22 (17–27)	17	8	25	68

Age is presented as median and interquartile range (IQR) due to non-normal distribution. Differences in age were primarily analyzed using the Kruskal–Wallis test (non-parametric). For robustness, a confirmatory ANOVA (parametric) was also performed, which yielded consistent results. TMJ pain prevalence was compared using the chi-square test. Kruskal–Wallis test *p*-value = 0.814; ANOVA test *p*-value = 0.81; Chi-square test *p*-value = 0.66.

**Table 3:** Comparison of oral function across four groups.

Parameter	OA (n = 27)	BDD (n = 41)	UDD (n = 27)	OC/DD (−) (n = 25)	*p*-Value	η^2^	OA vs. BDD	OA vs. UDD	OA vs. OC/DD (−)	BDD vs. UDD	BDD vs. OC/DD (−)	UDD vs. OC/DD (−)
Occlusal Contact Area (mm^2^)	10.36 ± 4.39	13.11 ± 5.97	15.49 ± 6.59	18.63 ± 10.6	<0.001	0.12	n.s.	n.s.	*	n.s.	n.s.	n.s.
Maximum Occlusal Force (N)	429.1 ± 151.77	536.05 ± 235.96	602.3 ± 239.12	708.52 ± 368.55	0.002	0.14	n.s.	n.s.	*	n.s.	n.s.	n.s.
Masticatory Efficiency (ΔE)	41.7 ± 5.43	41.18 ± 6.06	37.89 ± 8.6	41.30 ± 6.51	0.142	0.05	n.s.	n.s.	n.s.	n.s.	n.s.	n.s.
Tongue Pressure (kPa)	29.77 ± 9.28	28.99 ± 6.81	27.49 ± 10.26	33.39 ± 10.9	0.134	0.05	n.s.	n.s.	n.s.	n.s.	n.s.	n.s.
Lip-closing Force (N)	11.85 ± 6.43	10.48 ± 3.67	10.03 ± 2.66	11.17 ± 3.73	0.425	0.02	n.s.	n.s.	n.s.	n.s.	n.s.	n.s.

Data are presented as mean ± standard deviation. Statistical comparisons were performed using one-way ANOVA and Bonferroni’s post hoc tests. Statistical significance: *p* < 0.0083 (0.05/6) (*); n.s., not significant.

**Table 4:** Comparison of cephalometric measurements across four groups.

Parameter	OA (n = 27)	BDD (n = 41)	UDD (n = 27)	OC/DD (−) (n = 25)	*p*-Value	η^2^	OA vs. BDD	OA vs. UDD	OA vs. OC/DD (−)	BDD vs. UDD	BDD vs. OC/DD (−)	UDD vs. OC/DD (−)
① SNA (°)	81.16 ± 3.68	80.67 ± 2.79	81.53 ± 3.87	81.34 ± 2.86	0.726	0.01	n.s.	n.s.	n.s.	n.s.	n.s.	n.s.
② SNB (°)	76.73 ± 4.7	77.41 ± 5.05	80.16 ± 4.88	81.67 ± 3.93	<0.001	0.15	n.s.	n.s.	*	n.s.	*	n.s.
③ ANB (°)	4.43 ± 2.85	3.27 ± 3.73	1.37 ± 3.64	0.31 ± 2.83	<0.001	0.21	n.s.	*	*	n.s.	*	n.s.
④ Facial angle (°)	83.2 ± 4.73	85.07 ± 4.88	87.83 ± 4.2	88.57 ± 3.6	<0.001	0.18	n.s.	*	*	n.s.	n.s.	n.s.
⑤ FMA (°)	34.81 ± 7.44	32.79 ± 8.0	30.41 ± 5.07	25.71 ± 7.31	<0.001	0.17	n.s.	n.s.	*	n.s.	*	n.s.
⑥ Y-axis (°)	67.47 ± 4.95	66.10 ± 5.35	63.92 ± 4.48	62.87 ± 4.41	0.003	0.11	n.s.	n.s.	*	n.s.	n.s.	n.s.
⑦ Gonial angle (°)	124.77 ± 6.81	126.27 ± 6.47	126.29 ± 6.27	122.11 ± 8.83	0.098	0.05	n.s.	n.s.	n.s.	n.s.	n.s.	n.s.
⑧ FMIA (°)	53.19 ± 11.21	56.6 ± 11.86	61.71 ± 10	62.52 ± 10.32	0.007	0.1	n.s.	n.s.	n.s.	n.s.	n.s.	n.s.
⑨ IMPA (°)	92.01 ± 9.36	90.61 ± 8.39	87.89 ± 9.39	91.76 ± 10.65	0.357	0.03	n.s.	n.s.	n.s.	n.s.	n.s.	n.s.
⑩ U1 to SN (°)	104.56 ± 6.39	108.84 ± 7.85	108.54 ± 8.42	113.06 ± 9.25	0.003	0.11	n.s.	n.s.	*	n.s.	n.s.	n.s.
⑪ Occlusal plane to FH (°)	20.06 ± 4.91	18.43 ± 4.96	17.24 ± 4.77	14.78 ± 4.61	<0.001	0.13	n.s.	n.s.	*	n.s.	n.s.	n.s.
⑫ Overjet (mm)	5.04 ± 2.77	5.22 ± 4.15	2.48 ± 3.37	1.88 ± 3.52	<0.001	0.15	n.s.	n.s.	n.s.	n.s.	*	n.s.
⑬ Overbite (mm)	0.17 ± 3.05	1.55 ± 3.15	1.15 ± 3.31	1.07 ± 2.39	0.33	0.03	n.s.	n.s.	n.s.	n.s.	n.s.	n.s.

Data are presented as mean ± standard deviation. Statistical comparisons were performed using one-way ANOVA and Bonferroni’s post hoc tests. Statistical significance: *p* < 0.0083 (0.05/6) (*); n.s., not significant.

## Data Availability

All of clinical data are available in the Supplementary Materials.

## Supplementary Materials

The following supporting information can be downloaded at: https://www.mdpi.com/article/10.3390/jcm14144921/s1, Table S1: Results of tests on normality of data, Figure S1: Histogram for each evaluation item, File S1: All the data analyzed in this study.
