# Supplementary material for: Structural Features of the Temporomandibular Joint Evaluated by MRI and Their Association with Oral Function and Craniofacial Morphology in Female Patients with Malocclusion: A Cross-Sectional Study
Source: J Clin Med. 2025 Jul 11;14(14):4921. doi: 10.3390/jcm14144921 (PMC12294956; doi:10.3390/jcm14144921)
Supplement: Supplementary file 1 [file jcm-14-04921-s001.zip › Supplemental.pptx]

## Slide 1
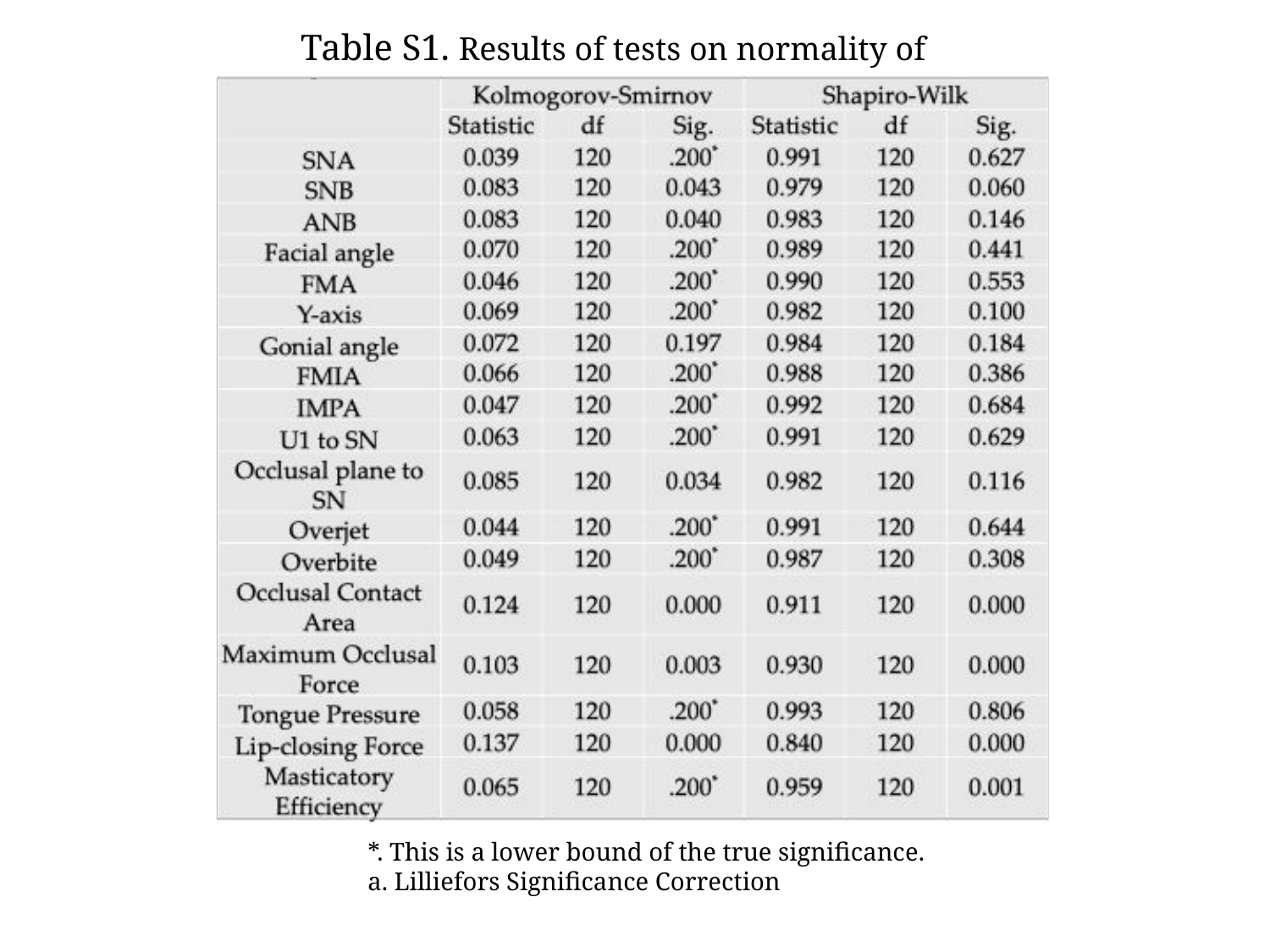

Table S1. Results of tests on normality of data
*. This is a lower bound of the true significance.
a. Lilliefors Significance Correction

## Slide 2
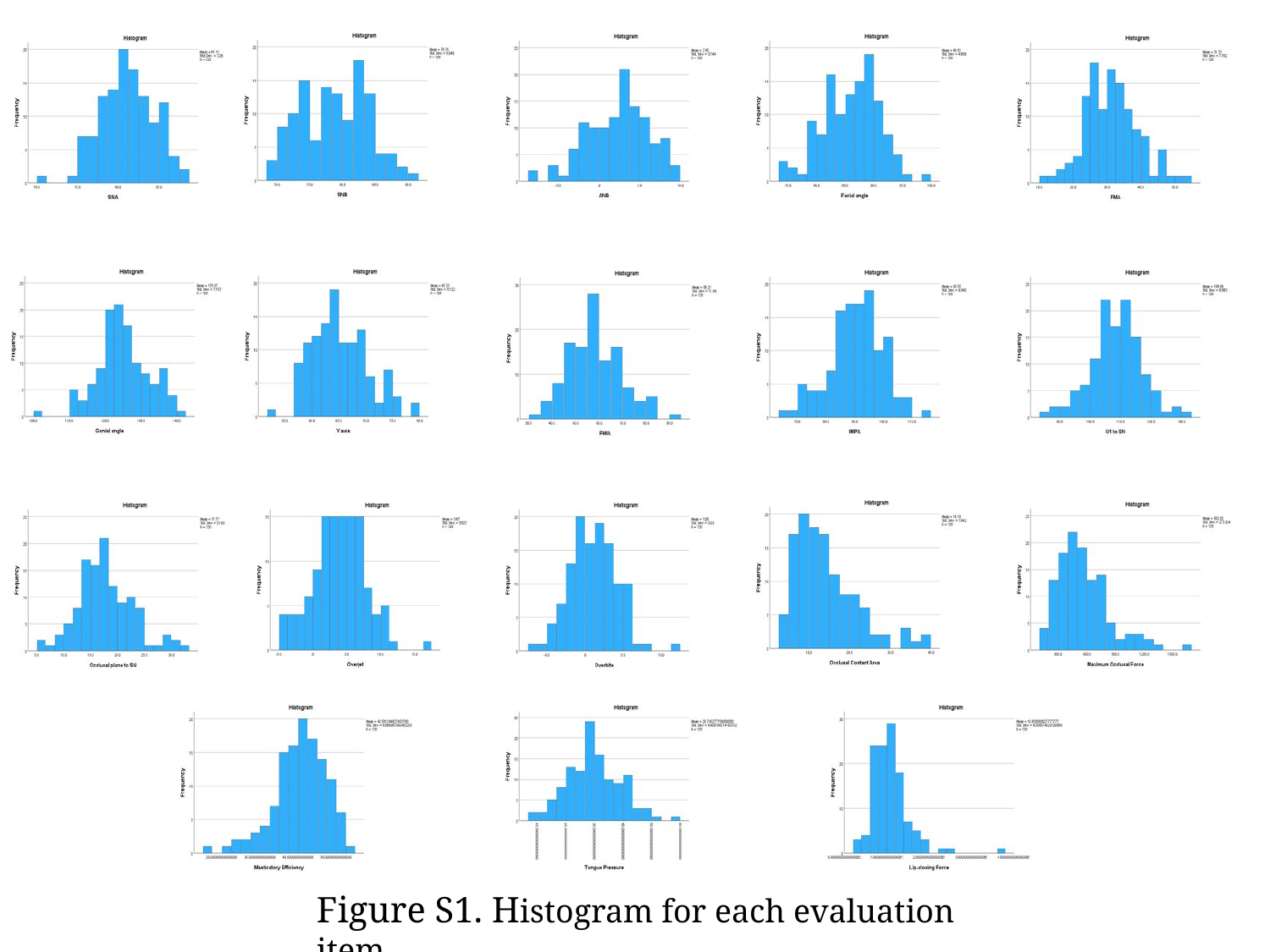

Figure S1. Histogram for each evaluation item
